# Amelioration of Photosynthesis and Quality of Wheat under Non-thermal Radio Frequency Plasma Treatment

**DOI:** 10.1038/s41598-018-30200-7

**Published:** 2018-08-03

**Authors:** Mahin Saberi, Seyed Ali Mohammad Modarres-Sanavy, Rasoul Zare, Hamid Ghomi

**Affiliations:** 1 Depatment of Agronomy, Faculty of Agriculture, Tarbiat Modares University, Tehran, Iran; 2 Iranian Research Institute of Plant Protection, Agricultural Research, Education and Extension Organization, Tehran, Iran; 3 Laser and Plasma Research Institute, Shahid Beheshti University, Tehran, Iran

## Abstract

Plasma treatment is recognized as a suitable technology to improve germination efficiency of numerous seeds. The objective of this paper is to demonstrate whether cold air plasma can change the quality and quantity of wheat yield. Effects of cold plasma treatment on wheat (Pishgam variety) yield were studied by a randomized complete block design experiment at the Faculty of Agriculture of Tarbiat Modarres University, Iran, during 2015–17. Seeds were pre-treated with 80 W of cold plasma at four levels of time, 60, 120, 180 and 240 seconds. Plasma effects on yield and quality of wheat were determined by measuring plant photosynthesis, grain yield, biological yield, 1000-grain weight, total chlorophyll, carotenoid, anthocyanin, protein and starch content. Results showed that plasma treatments had positive effects on wheat characteristics, and treatment of 180 s had the highest stimulatory effect. In both years, cold plasma increased grain yield at 180 s, but decreased it at 240 s compared with control. The rate of plant photosynthesis, grain yield, 1000-grain weight, carotenoid and anthocyanin were enhanced at 180 s. The starch content and grain protein were enhanced at 120 s cold plasma application compared with control.

## Introduction

Wheat (*Triticum* spp.) is a major crop worldwide and is considered a strategic crop in several countries. Crop yield is highly dependent on seed quality, for example seed germination, vigour and size. Seed quality is an essential factor for crop production and food security particularly by increasing the effects of climate change^1^. Priming is a seed enhancement method that might improve seed performance and increase plant quality and quantity. It is demonstrated that on-farm seed priming markedly improved establishment and early vigour of seedlings, resulting in faster development, earlier flowering and maturity and higher yield^2^ as also improved emergence, yield and quality was observed in direct-seeded rice^3^. The positive effect of rice seed priming to is attributed to an increase in endospermic amylase activity resulting from the ameliorated soluble sugar content of the primed seed^4^. It is reported that seed priming could be used to achieve higher seed vigour and seedling growth in maize^5^.

In the past ten years, atmospheric-pressure plasmas have emerged as a competitive mean to other methods of enhancing seed and seedling vigour and disease resistance. Plasma (ionized gas) as a new technique for seed priming, is one of the four fundamental states of matter, and was first described by chemist Irving Langmuir in the 1929^6^. Plasma is electrically conductive and can be produced in the lab by applying a high voltage between two electrodes. An important property of plasma is its temperature as it can be very hot (5000 to 20,000 K) or non-thermal plasma (300 K). The non-thermal plasma induces physical and chemical reactions and is suited for living tissues of animals and plants (that are sensitive to the high temperature)^7^. Recently, the non-thermal plasma is used as a viable alternative to replace traditional methods namely seed treatment scarification, heat treatment and chemical treatment^8^. The cold plasma treatment is thought to be a fast, economic and pollution-free method to improve seed performance, plant growth and ultimately plant production^8,9^. This treatment plays a crucial role in a broad spectrum of plant development and physiological processes in plants, including the promotion of seed germination and seedling growth^10,11^, activation of photosynthesis^12,13^, and regulation of carbon and nitrogen metabolism^14,15^. In safflower, compared with the control, there was significant increase in germination rate (50%) and reduced germination time by 24 hours^8^. It is shown that 80 W of cold plasma treatment significantly increased tomato nitrogen (N) and phosphorus (P) absorption by 12.7% and 19.1%, respectively, and markedly ameliorated tomato root morphology, and root length, surface area and volume^16^. Also germination potential and rate of peanut were markedly raised by 150% and 21%, respectively by the plasma application^13^.

## Results

### Biological yield

There was a significant difference between years and treatments for wheat biological yield, but there was no significant difference between interaction of T*Y (Table 1). Treatments 60 s and 180 s in 2016 were significantly higher for biological yield compared with control (Table 2). In 2017, significant differences was obtained in 60 s, 120 s and 180 s treatments compared with control (Table 2).

**Table 1:** Analysis of variance of characteristics in wheat under levels of plasma treatment.

Source	df	Biological yield	Mean square	1000 grain weight
			Grain yield	
Y	1	4324175.53**	260736.34**	235.20**
R(Y)	4	280543.71	13230.92	8.06
T	4	677069.91**	58468.14**	20.21
T*Y	4	125287.86	31485.96**	10.28
Error	16	78301.23	5038.82	9.52
Cv(%)		14.38	12.08	9.66

Notes: * and ** Significant at 5% and 1% probability levels, respectively. Y, year; R(Y), replication year; T, treatments; T*Y, interaction between treatments and years; d.f, degree of freedom; CV, coefficient of variation.

**Table 2:** Mean comparison of effect of cold plasma on characteristics of wheat in the field.

Treatments	Year	Biological yield (gm^−2^)	Grain yield (gm^−2^)	1000 grain weight (g)
Control	2016	1980.0b	622.33b	32.00b
	2017	1285.83c	469.73b	27.00a
60 s	2016	2773.3a	764.80ab	34.00ab
	2017	1857.87a	515.87ab	28.33a
120 s	2016	2403.3ab	724.53ab	35.00ab
	2017	1681.20ab	523.47ab	29.33a
180 s	2016	2740.3ab	842.0a	39.33a
	2017	1618.93	595.47a	31.00a
240 s	2016	1726.7b	450.13c	33.33ab
	2017	1383.27c	367.00c	30.00a

Note: Means in each column followed by similar letter(s) are not significantly different at 5% probability level using Duncan Multiple Rang Test.

### Grain yield

There was a significant difference between the years and treatments and interaction between them for wheat grain yield (Table 1). In both years the 180 s treatment significantly increased the grain yield by 31.62% compared with control (Table 2).

### 1000-grain weight

There was a significant difference between years, but no significant difference between treatments and interactions between them (Table 1). The highest seed weight was observed in 180 s treatment but only significant in 2016, and which produced 19.20% more 1000-grain weight than the control (Table 2).

### Stomatal conductance

There was a significant difference between years and treatments and interaction between T*Y (Table 3). Treatment 180 s, significant differences was obtained compared with control but only significant in 2017 (Table 4).

**Table 3:** Analysis of variance of characteristic under level of plasma treatment.

Mean square					
Source	df	Stomatal conductance	Mesophyll conductance	Sub-stomatal CO_2_	Net Photosynthesis rate
Y	1	0.00033*	0.00059*	43.583*	0.29
R(Y)	4	0.00003	0.00004	6.17	0.60
T	4	0.00011*	0.00029**	11.62**	7.91**
T*Y	4	0.00015**	0.00012**	1.39	0.50
Error	16	0.00002	0.00002	1.35	0.34
Cv(%)		14.15094	13.15787	14.27	9.06

Notes: * and ** Significant at 5% and 1% probability levels, respectively. Y, year; R(Y), replication year; T, treatments; T*Y, interaction between treatments and years; d.f, degree of freedom; CV, coefficient of variation.

**Table 4:** Mean comparison of effect of cold plasma on characteristics of wheat in the field.

Treatments	Year	Stomatal conductance (mol m^−2^ s^−1^)	Mesophyll conductance (mol m^−2^ s^−1^)	Sub-stomatal CO_2_ (pa)	Net Photosynthesis Rate (µmol m^−2^ s^−1^)
Control	2016	0.040a	0.025b	5.11b	5.02d
	2017	0.030bc	0.024b	8.82b	5.50b
60 s	2016	0.040a	0.029ab	6.13b	7.17ab
	2017	0.033ab	0.046a	8.30b	7.09a
120 s	2016	0.040a	0.030ab	7.22ab	6.40bc
	2017	0.033ab	0.050a	9.23b	7.30a
180 s	2016	0.046a	0.034a	9.87a	7.73a
	2017	0.040a	0.045a	11.07a	8.06a
240 s	2016	0.026b	0.032ab	6.38b	5.68cd
	2017	0.023c	0.030b	9.35b	5.05b

Note: Means in each column followed by similar letter(s) are not significantly different at 5% probability level using Duncan Multiple Rang Test.

### Mesophyll conductance

There was a significant difference between years, treatments and the interaction between them for mesophyll conductance (Table 3). In 2016, only 180 s treatment caused a significant increase in mesophyll conductance compared with control (Table 4), but in 2017, treatments 60 s, 120 s and 180 s caused a significant increases compared with control (Table 4).

### Sub-stomatal CO_2_

There was a significant difference between years and treatments for sub-stomatal CO_2_ of wheat, but there was no significant difference for interaction of T*Y (Table 3). In both years, 180 s treatment had a significant difference compared with control (Table 4).

### Net photosynthesis rate

There was no significant difference between years and interaction of T*Y for net photosynthesis rate, but a significant difference was observed between treatments (Table 3). In both years, 60 s. 120 s and 180 s treatments significantly increased net p/s rate compared to the control (Table 4).

### PAR (photosynthetically active radiation)

There was a significant difference between treatments and interaction between years and treatments (Table 5). In 2016, 180 s significantly increased PAR compared to control (Table 6). In 2017, all plasma treatments significantly increased PAR compared to the control (Table 6). In both years the 180 s treatment significantly increased the PAR by 30% compared with control (Table 6).

**Table 5:** Analysis of variance of characteristic in wheat under level of plasma treatment.

Source	Mean square				
	Df	PAR	Carotenoids	**A**nthocyanins	**T**otal Chlorophyll
Y	1	724.32	9.48**	7.89*	0.74**
R(Y)	4	40666.74	0.38	1.02	0.01
T	4	110434.83**	4.18**	1.17**	0.05
T*Y	4	39981.56*	0.45	0.38	0.05
Error	16	13639.18	0.20	0.21	0.04
CV(%)		7.84	11.65	10.87	16.70

Notes: * and ** Significant at 5% and 1% probability levels, respectively. Y, year; R(Y), replication year; T, treatments; T*Y, interaction between treatments and years; d.f, degree of freedom; CV, coefficient of variation; PAR, (photosynthetically active radiation).

**Table 6:** Mean comparison of effect of cold plasma on characteristics of wheat in the field.

Treatments	Year	PAR (µmol m^−2^ s^−1^)	Carotenoid (µmol ml^−1^)	Anthocyanin (µmol ml^−1^)	Total Chlorophyll (µmol ml^−1^)
Control	2016	1363.30b	2.05c	2.89c	0.89b
	2017	1257.75c	3.70c	4.17b	0.89c
60 s	2016	1481.70ab	3.34b	4.03ab	1.51a
	2017	1469.34b	4.07bc	4.61ab	1.15ab
120 s	2016	1557.90ab	2.80bc	3.53ab	1.24a
	2017	1519.65b	4.46b	4.97ab	1.26a
180 s	2016	1667.60a	4.60a	4.55a	1.50a
	2017	1727.41a	5.75a	5.22a	1.08b
240 s	2016	1451.30ab	3.71ab	3.66ab	1.54a
	2017	1496.73b	4.13bc	4.82ab	1.22ab

Note: Means in each column followed by similar letter(s) are not significantly different at 5% probability level using Duncan Multiple Rang Test. PAR, (photosynthetically active radiation).

### Carotenoid

There was a significant difference between years and treatments, but there was no significant interaction between years and treatments (Table 5). In 2016, 60 s, 180 s and 240 s significantly increased carotenoid compared to the control (Table 6). In 2017, 120 s and 180 s significantly increased carotenoid compared to the control. The 180 s treatment significantly increased the carotenoid vs control in both years by 80% (Table 6).

### Anthocyanin

There was a significant difference between years and treatments for anthocyanins of wheat, but there was no significant interaction between years and treatments (Table 5). The 180 s treatment significantly increased the anthocyanin vs control in both years by 38.38% (Table 6).

### Total chlorophyll

There was a significant difference between years, but no significant difference between treatments in the total chlorophyll of wheat. There was no significant difference between interaction treatment effects and years (Table 5). In both years, the effect of all plasma treatments on the amount of chlorophyll was significant compared with control (Table 6).

### Protein

No significant difference was recorded between treatments in the protein of wheat, but there was a significant difference between years and interaction between years and treatments (Table 7). Significant results vs control were obtained by 60 s and 180 s (2016) and 120 s (both years). That was 49% increase for average of both years, 120 s vs control (Table 8).

**Table 7:** Analysis of variance of characteristics in wheat under levels of plasma treatment.

Mean square			
Source	df	Protein	**S**tarch
Y	1	0.0036*	0.02
R(Y)	4	0.0001	0.54
T	4	0.0002	2.44*
T*Y	4	0.0008**	1.62
CV(%)		16.2601	6.92

Notes: * and ** Significant at 5% and 1% probability levels, respectively. Y, year; R(Y), replication year; T, treatments; T*Y, interaction between treatments and years; d.f, degree of freedom; CV, coefficient of variation.

**Table 8:** Mean comparison of effect of cold plasma on characteristics of wheat in the field.

Treatments	Year	Grain Protein (%)	**G**rain Starch (mg/10 g)
Control	2016	6.3b	10.17a
	2017	5.3b	10.53bc
60 s	2016	10.0a	11.21a
	2017	5.6ab	9.82c
120 s	2016	10.0a	11.15a
	2017	7.3a	12.51a
180 s	2016	8.3ab	11.60a
	2017	6.0ab	11.10bc
240 s	2016	7.0b	11.24a
	2017	6.3ab	11.68ab

Note: Means in each column followed by similar letter(s) are not significantly different at 5% probability level using Duncan Multiple Rang Test.

### Starch

There was no significant difference between years, but there was a significant difference between treatments in the starch of wheat. There was no significant interaction between treatment effects and years (Table 7). The starch content was increased by 14.92% in 120 s treatment which was significant compared with the control but only significant in 2017 (Table 8).

## Discussion

The present study showed that cold plasma had a positive effect on the characteristics of the quantity and quality of wheat. In both years of 2016–17, the 180 seconds cold plasma treatment produced the highest stimulatory effect among different treatments; in 180 s yield of the treated wheat was 7.10 t.ha^−1^, 31.62% higher than control. The effect of the increased plasma yield was similar to the result obtained in soybean where cold plasma of a power of 80 W increased germination rate by 14.66% and vigour was increased by 63.33%^11^. Plasma by restructuring, increases the permeability of the seed coat, stimulates seed germination in plants, such as *Chenopodium album*^17^, *Oryza sativa*^13^, *Triticum aestivum*^12^, and *Solanum melongena*^15^ causing an increase in the performance of the seed. The researchers also concluded that the plasma treatment accelerates the decomposition of carbohydrates and soluble proteins^18,19^. Many studies show that soluble carbohydrate is closely related to photosynthesis and yield^20^. Carbohydrate is the main product of photosynthesis and plays an impressive and dramatic role in plant metabolism^1^.

Plasma has improved relationship between cells and enzymes, such as alpha-amylase and protease which play an important role in the germination process^18,21^. It was shown that the activity of peroxidase and catalase enzymes slightly increased following plasma treatment^22^. It is also reported that seed treated by plasma significantly increased the superoxide dismutase activity in maize^14^. Similar result was obtained for tomato^23^.

In our research, we found a positive correlation between photosynthesis rate and stomatal conductance (R^2^ = 0.67*). The positive relationship could be due to increased stomatal conductance and CO_2_ level input and as a result enhancement of chlorophyll^1^. We also found a positive correlation between chlorophyll and photosynthesis rate (R^2^ = 0.91**). Enhanced leaf photosynthesis increased the flow of materials in plants^24^ resulting in more food supply for grain filling and subsequently increase the plant performance^25^. Koc *et al*. (2003) indicated a high correlation between stomatal conductance and photosynthesis rate per unit leaf area^26^. Using cold helium plasma at the booting stage, it was observed that the treated wheat had more length and stronger roots as the key factors to increase absorption of water and nutrition^27^. Due to plasma treatment the plant height is increased and as the result the plant could compete for more sunlight. Higher level of absorption of PAR (photosynthetically active radiation) causes higher stomatal conductance, leaf area and chlorophyll content^27^. On the other hand the absorption level of PAR causes osmolite accumulation such as soluble sugars and carbohydrates^1^. At 180 s, absorption level of PAR was increased by 30% compared with the control (Table 6) which in turn increased photosynthesis rate and yield. Other researchers have also focused on the effects of plasma enhanced chlorophyll^14,17^.

The 240 s treatment reduced grain yield compared to the control (Table 1). It was demonstrated that longer time of plasma treatment of spring wheat reduced the germination down to 70% which was due to the damage of seeds inflicted by plasma bombardment^28^. It was indicated that the exposure of seeds to plasma for a longer time had adverse effect on the seed yield^29^. This is an expected effect due to the highly oxidative character of the excited species in air plasmas^30^. The increase of reactive oxygen species (ROS) by longer plasma treatment is also reported^31^. In all aerobic organisms the production of reactive oxygen species (ROS) takes place during normal metabolic processes^32^. When the amount of free radicals (ROS) in the cell is more of antioxidants, damage to cells and tissues is certain. The ROS activity causes lipid peroxidation, the changing nature of proteins, single-strand breaks of DNA, and the proteins to be cross-linked^33^.

**Figure 1:**
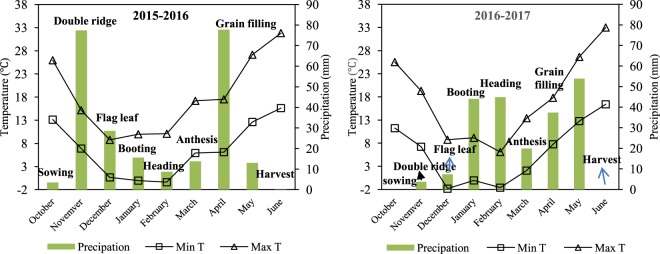
Monthly maximum and minimum air temperatures (°C), and precipitation (mm) recorded during the growing season in 2015–2016 and 2016–2017.

Wheat grain yield and yield components in 2016 were greater than that of 2017 which is mostly attributed to the weather conditions of the two years. In 2016 during grain filling there was 40 mm increase in rainfall and decrease of 2 °C temperature (Fig. 1) causing longer growth period and increased chlorophyll content and Net Photosynthesis Rate compared with 2017. This was in concordance with Mokhtassi-Bidgoli *et al*. (2013) results^34^.

The results indicates the ameliorated amount of anthocyanin and carotenoid by 38.38% and 80% respectively, at 180 s plasma compard to the control (Table 6). Changes occurring in the seed coat after plasma treatment, enhance hydrophilic ability of the seeds, improving water uptake by the seed, thus increasing the amount of assimilates and transferred reserves^11^. Reduction of seed reserves is the most important factor determining the efficiency and initial growth and development of the seedling^35^. A higher transferred reserves potential in wheat is mostly dependent on the amount of assimilates that resulting in greater seed weight on mother plant^36,37^. Under stress condition seed yield is dependent on the amount of assimilants^38^. Therefore, maximum concentration of anthocyanin seems to be conditioned by the availability of assimilates^39^. Seed treatment with cold plasma results in the accumulation of secondary metabolites such as anthocyanins and carotenoids, increasing their immunity to abiotic and biotic stresses, such as drought plants^40^ and diseases^41^.

Wheat quality is primarily a function of the quality and quantity of seed protein. In this study 120 s plasma treatment increased protein content by 49%. It is reported that soybean protein was increased by 25.08% due to plasma treatment^11^. Similar results were obtained in protein of corn^41^ and brown rice^13^. It was explained that enzymatic proteins such as amylase and protease are two direct metabolic factors in the seed^23^. The breakdown of fatty acids during germination of the seeds increased the amount of protein^42^. Also Jiang *et al*. (2018), showed that 80 W of cold plasma treatment significantly increased absorption of nitrogen (N) by 12.7%, which is an important factor increasing protein content^16^.

In this study 120 s plasma treatment increased wheat starch by 14.92%. Starch carbohydrate is the most important constituent of wheat. Although wheat has many applications, the main root of its popularity is due to its capacity to produce bread. The structure and composition of the grain in the production of a good bread are very effective. Presumably, the moment and extent of starch gelatinization and the concomitant water migration influence the structure formation during baking^43^. Starch content, being responsible for greater water absorption, facilitates the bread-making process through enhanced fermentation of the sugar into CO_2_ and water^44,45^.

## Methods

The experiment was carried out in the experimental field of the Agricultural Research Station of Tarbiat Modarres University, Tehran, Iran (35°41′ N, 51°10′ E, Altitude 1215 m). Seeds were sown on 8 October 2015 and 9 October 2016 and data were recorded on 28 July 2016 and 9 July 2017. Seeds (Pishgam variety) of wheat (*Triticum aestivum* L.) were obtained from Seed and Plant Improvement Institute (Karaj, Iran). Seeds were kept under ambient condition (5 °C and humidity below 60%) until planting.

**Figure 2:**
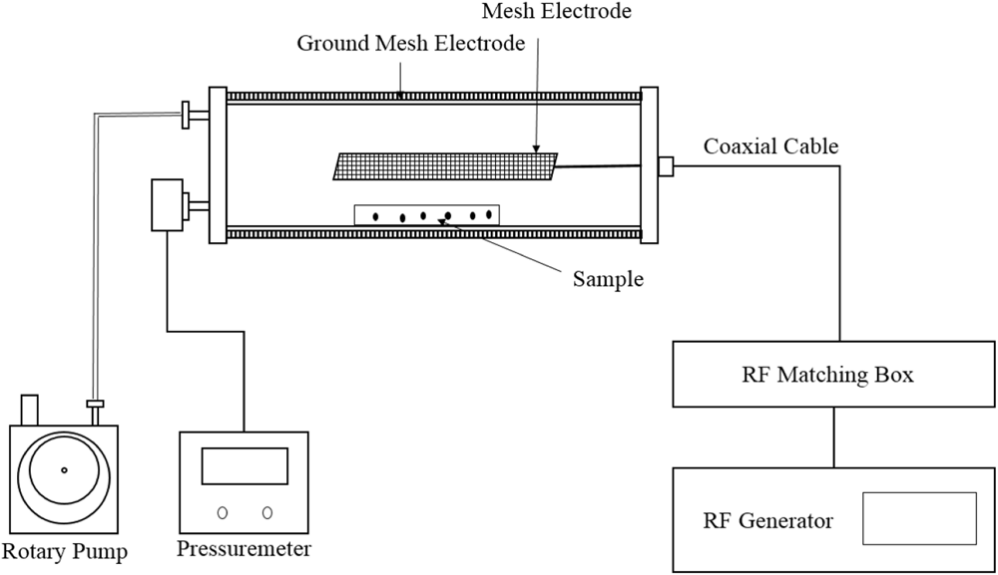
Process of treating wheat seeds by the RF plasma device.

The experimental setup has been shown in Fig. 2. The plasma has been applied by radiofrequency (RF) plasma reactor operated with air at 13.56 MHz. The vacuum chamber has been made by a cylindrical Pyrex tube with inner diameter 80 mm and 300 mm in length. The outside of the Pyrex tube has been grounded by metallic mesh. The aluminum power electrode has been fixed at the center of cylinder (50 mm in width and 100 mm in length). The sample has been placed over the Pyrex tube. The gap between the power electrode and the sample is 40 mm. The plasma has been generated at 0.1 mbar by air gas at 80 W. Wheat seeds were treated for 0 (C, control) 60, 120, 180 and 240 s.

Each plot consisted of 20 rows, spaced at 30 cm and a length of six meters (400 plant per m^2^). Seeds were planted at a depth of 5 cm manually. Drip irrigation was applied according to soil moisture. Weed control and management of pests and diseases were conducted throughout the plant growth period. Textures and elements of the soil were determined before planting. The adequate nitrogen, phosphorus and potassium were provided for plants according to soil test. The experiment was conducted in a randomized complete block design with three replications for two cropping seasons. In each plot, samples for grain yield and yield components were harvested in the middle of the plot to avoid any border effects. Data were analyzed using SAS (9.02)^46^. The effects of treatments were determined by analysis of variance (ANOVA). Duncan Multiple Rang test was used to measure statistical differences between treatments and control.

CO_2_ gas exchange measurements of photosynthesis in units of micromoles per square meter per second and stomatal conductance (mole per square meter per second) were performed on flag leaf at 10 days after pollination using photosynthesis meter model LI-COR 6400XT Version 6 (Lincoln, Nebraska). The content of chlorophyll, anthocyanins and carotenoids were measured on the flag leaf^47^. 0.1–0.2 g of fresh leaf sample was extracted in 2 ml of 15% Tris buffer and 85% acetone solution, then samples were centrifuged for 3 minutes at a rate of 12000 rpm. The absorption spectrums (spectrophotometrically) were measured at 470, 537, 647 and 663 nm. The results were calculated according to the method of Krizek *et al*.^48^.

In order to measure protein content Kjeldahl method was used^49^. Approximately 1 g of milled seed was hydrolyzed with 15 ml sulphuric acid (H_2_SO_4_) containing two copper catalyst tablets in a heat block at 430 °C until 2 h. After cooling, 50 ml distilled water +30 ml NaOH was added to the hydrolysates for 4 min for neutralization prior to titration. After titration the amount of total nitrogen in the raw materials were multiplied with the factor of 6.25 in order to determine total protein content. Total starch content was analyzed using the Megazyme total starch analysis (AA/AMG) procedure^50^. A 100 mg of milled grain was wet with 0.2 ml of ethanol and treated with thermostable a-amylase to partially hydrolyze the starch. After completely dissolving the starch, dextrins were quantitatively hydrolised to glucose by amyloglucosidase. The amount of glucose was measured and the starch content was estimated as described by McCleary *et al*. (1994).

## Conclusion

This paper has focused on the quantity and quality increase of wheat yield by treatments of seeds by air cold plasma. The results of two years of study showed that significant yield increase was achieved following treatment after 180 s exposure of seeds with cold plasma. Quantitatively, plasma treatment of 180 s resulted in increased grain yield by 31.62% which is an important step to feed the growing population on earth. Qualitatively, plasma treatment of 120 s resulted in starch and protein content by 49% and 14.92% respectively, compard to control.
